# Feeding Preferences and the Nutritional Value of Tropical Algae for the Abalone *Haliotis asinina*

**DOI:** 10.1371/journal.pone.0038857

**Published:** 2012-06-14

**Authors:** Alex R. Angell, Igor Pirozzi, Rocky de Nys, Nicholas A. Paul

**Affiliations:** School of Marine and Tropical Biology & Centre for Sustainable Tropical Fisheries and Aquaculture, James Cook University, Townsville, Australia; University of Connecticut, United States of America

## Abstract

Understanding the feeding preferences of abalone (high-value marine herbivores) is integral to new species development in aquaculture because of the expected link between preference and performance. Performance relates directly to the nutritional value of algae – or any feedstock – which in turn is driven by the amino acid content and profile, and specifically the content of the limiting essential amino acids. However, the relationship between feeding preferences, consumption and amino acid content of algae have rarely been simultaneously investigated for abalone, and never for the emerging target species *Haliotis asinina*. Here we found that the tropical *H. asinina* had strong and consistent preferences for the red alga *Hypnea pannosa* and the green alga *Ulva flexuosa*, but no overarching relationship between protein content (sum of amino acids) and preference existed. For example, preferred *Hypnea* and *Ulva* had distinctly different protein contents (12.64 vs. 2.99 g 100 g^−1^) and the protein-rich *Asparagopsis taxiformis* (>15 g 100 g^−1^ of dry weight) was one of the least preferred algae. The limiting amino acid in all algae was methionine, followed by histidine or lysine. Furthermore we demonstrated that preferences can largely be removed using carrageenan as a binder for dried alga, most likely acting as a feeding attractant or stimulant. The apparent decoupling between feeding preference and algal nutritive values may be due to a trade off between nutritive values and grazing deterrence associated with physical and chemical properties.

## Introduction

Understanding feeding preferences of abalone is integral to developing sustainable diets for their aquaculture because of the expected link between preference and performance [1], [2]. However, preferences between abalone species are distinct and diverse for those from different geographic regions, making it difficult to generalise which algae offer the best nutrition for new species development. For example, Australasian (*Haliotis rubra*, *H. laevigata* and *H. roei*) and tropical Western Pacific species of abalone (*H. asinina*) prefer red algae (e.g. *Asparagopsis armata* and *Gracilaria* sp.) over brown algae (e.g. *Sargassum fallax* and *Ecklonia radiata*) [3]–[6]. In contrast, abalone from North and Central America (*H. rufescens, H. fulgens* and *H. corrugata*) [7], Japan (*H. discus hannai* and *H. diversicolor supertexta*) [8], New Zealand (*H. iris*) [9] and South Africa (*H. midae*) [10]–[13] prefer brown algae over red and green algae. This variability suggests that the diverse preferences of abalone are influenced by multiple factors, including secondary metabolites or chemical defences [5], [14], [15], the toughness or physical defence of the algae [16], [17], and nitrogen or protein content [11], [18].

Nitrogen, and more specifically protein content, plays an important role in determining preferences of abalone [11], [18], and marine herbivores more generally [19], [20], [21], especially amongst algae that have limited physical or chemical defences (e.g. [18]). Furthermore, nitrogen (as protein) is the most limiting nutrient for growth of any herbivore [22], and is the crucial component in diet formulation of abalone [23]. The importance of protein for abalone is highlighted by the large differences between the optimal protein content in artificial diets, ranging from 27% up to 47% [24]–[27], and the protein composition of the natural algal diet. Algal protein composition is highly variable between species, ranging from 1.1 to 39% [28], [29], and within species, based on environment, season and age [30], [31]. Therefore, natural algal diets do not appear to provide a consistent dietary source of protein.

In addition to the quantity of protein in a diet, the quality of this protein in terms of its amino acid profile is critical in optimising growth and contributes to how efficiently an animal utilises feed [32]. However, the links between protein quantity and protein quality are not well established for abalone, and the use of high protein diets to optimise high growth rates (e.g. 40–45%; [27], [33]) suggests that protein is not well utilised. Notably, animals do not have a requirement for protein *per se* but rather the amino acids from which they are created, and it is the first limiting essential amino acid (relative to the requirement of the animal) that determines the “effective” protein content, i.e. the adjusted amount which potentially can be used for growth. Differences in the degree of limitation in diets will contribute to the variability in optimal reported dietary protein content [24], [25], [27], [34], [35]. However, no study has simultaneously compared protein content and amino acid profile of algal diets to the feeding preferences of abalone to test the strength of the relationship between preferences and nutritional value.

Therefore, the primary aim of this study is to quantify the feeding preference hierarchy of the tropical abalone *H. asinina*, a developing aquaculture species, and examine the links between preferences, crude protein contents and amino acid contents. A preference hierarchy of algae was first developed, using both multiple choice assays and consumption rates in a no-choice assay. Secondly, the nutritional value of these algae were deconstructed, by quantifying water content, carbon and nitrogen content, and both protein and amino acid concentrations. These compositional data, along with established physical and chemical properties of the algae, are discussed in relation to the preference hierarchy with a focus on the effective protein content in relation to limiting amino acids. Finally, carrageenan was used as means to bind dried algae in a standardised diet to test whether the preference hierarchy of *H. asinina* can be modified to enhance the consumption of highly nutritious but lower preference species.

## Materials and Methods

### Study Organisms

Tropical abalone *Haliotis asinina* were collected from Batt Reef (16°24′ S, 145°46′ E), Great Barrier Reef at ∼3 m under Great Barrier Reef Marine Park (GBRMPA) permit number G10/33487.1. Abalone were maintained in an outdoor recirculating system at James Cook University with water temperature ranging from 24.5 to 32°C (mean 26.5°C) and were fed a mixed diet *ad libitum*. Abundant brown, red and green algae were collected from shallow reefs at Nelly Bay, Magnetic Island (19°09′55′′ S, 146°51′02′′′ E) 1–2 days prior to feeding assays under GBRMPA permit number GO6/20234.1. The brown algae used in this study were *Cystoseira trinodis* (Forssk.) C. Agardth, *Padina australis* Hauck and *Sargassum flavicans* var. *moretonense* Grunow. The red algae used were *Asparagopsis taxiformis* (Delile) Collins and Harvey, *Hypnea pannosa* J. Agardh, *Jania crassa* J.V. Lamouroux and *Laurencia majuscula* (Harvey) Lucas. The green alga used was *Ulva flexuosa* (Wulfen *ex* Roth) J Agardh. All algae come from distinct genera and hereafter are referred to by genus.

### Feeding Assays

The feeding preferences hierarchy of *H. asinina* was evaluated using two types of assay. Firstly, choice assays quantified preferences in a multiple choice scenario. Secondly, no-choice assays quantified the consumption rates of each species independently. Both choice and no-choice feeding assays were conducted in an outdoor recirculating system (mean temperature of 26.5°C). Experimental units were constructed by placing a 3 L treatment (feeding) container inside a 7 L (autogenic control) container [36]. This system provided a continuous flow of water from the inner treatment container into the outer control container. All algal portions used in the assays were healthy and there was no degradation in either controls or treatments during the experiments.

### Choice Feeding Assays

Two choice feeding assays (A and B) assessed preference hierarchies amongst available algae at each time; *Asparagopsis* (A), *Hypnea* (A & B), *Laurencia* (A), *Jania* (B), *Cystoseira* (A & B), *Padina* (B), *Sargassum* (A & B) and *Ulva* (A). Prior to each assay, individual algae were excised into two equal portions, blotted dry and weighed. One portion was secured to the rim of the treatment container and the other to the control container. The amount of algae was roughly standardised to volume rather than weight (typically ∼30 cm^3^, on average), as the water content varied between species (see Results). This was done to ensure that abalone had an equal chance of contact with each species. Abalone were weighed prior to each assay (n = 11 & 19 abalone in A & B, respectively, 1 abalone per replicate). Abalone ranged from 35–80 g (A) and 29–184 g (B).

Individual animals were monitored and a replicate was stopped and weighed once the abalone had eaten a large proportion of an alga (a majority, >50% by sight), or after 48 hours. Remaining treatment and control algae were blotted dry and weighed. Time (hours) was used to calculate daily feeding rates. Most feeding was completed in less than 1 day. Preferences for each replicate were determined by the consumption of each species (W) calculated using the following formula which adjusts for any weight change in the autogenic controls (WC).


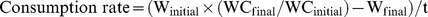
 Consumption rates were standardised for abalone size (body weight, BW) to 100 g (i.e. the mean abalone size in assay B) for graphical representation as g FW algae 100 g^−1^ BW day^−1^.

### No-choice Feeding Assay – Natural Diet

All the algae species that were tested in the choice feeding assays were subsequently used in a no-choice assay. The same experimental system and protocol were used as in the choice feeding assay, with the exception that an individual alga was secured to the containers and replicates were left for a longer period (up to 4 days) if more than ∼50% of the biomass (as before) had not been consumed. All remaining algae were collected, dried and reweighed and consumption rates calculated as per choice assays.

The influence of size on feeding rates (g day^−1^, Eq. 1) was formally compared with Analysis of Covariance (ANCOVA) (see Statistical Analysis) using similar sized abalone for each treatment (n = 5 abalone per algal species, 40 abalone in total ranging from 45 g to 140 g). Graphical representation of the no choice data is provided in a consistent format to the choice assays: g FW algae 100g^−1^ BW day^−1^.

### Wet: Dry Weight, Nitrogen and Carbon Compositional Analysis

Five replicates of each algal species used in the feeding assays were analysed for composition. Samples were washed of any epiphytes, blotted dry and the wet weight determined. Samples were subsequently dried at 50 °C for 48 hours until no further weight change. The nitrogen and carbon content of each alga was quantified using a Carlo-Erba elemental analyser by the Research School of Biological Sciences, Australian National University, Canberra. The carbon: nitrogen ratio was then calculated.

### Protein and Amino Acid Analysis

A mixed sample of 5–10 individuals from each species was collected from the field, washed of any epiphytes, freeze dried and milled. A sub-sample of 1 g was then analysed for amino acids. The amino acids analysed were aspartic acid/asparagine, glutamic acid/glutamine, serine, histidine, glycine, threonine, cysteine/cystein, alanine, taurine, arginine, tyrosine, valine, methionine, phenylalanine, isoleucine, leucine, lysine, proline and tryptophan. Amino acids were analysed using pre-column derivitised HPLC (ChemCentre, Bentley, Western Australia, and, the Australian Proteome Analysis Facility, Macquarie University, Sydney - for tryptophan).

Protein content was calculated as the sum of the amino acids for each species. The first essential limiting amino acid for each alga was determined as the lowest relative proportion to the published values for amino acid composition of *H. asinina* muscle tissue (see Table 1). When limited or no experimental data is available, initial estimates of amino acid requirements are typically derived from whole-body or muscle tissue content. However, amino acids deposited into the body represent a minor proportion of digested amino acids and thus muscle tissue composition can potentially overestimate amino acids that are preferentially deposited (e.g. lysine and leucine) and underestimate those with high metabolic turnover (e.g. methionine, threonine, histidine and arginine) [37]. However, experimental data for many cultured animals, including carp [38] and catfish [39], have shown that body tissue analysis is a sufficient indicator of amino acid requirement. Thus composition analysis currently remains an accepted proxy for amino acid requirement for species with limited nutritional information [37]. For *H. asinina* the available protein content of each alga was adjusted according to the level at which these first limiting amino acids were supplied. This was done by multiplying the level of the first limiting amino acid (%) by the protein content of each alga to indicate the biologically available protein for growth. The essential amino acid index (EAAI, Eq. 2) was subsequently calculated for each alga to examine protein quality, more generally, relative to the requirements of *H. asinina* [40].

**Table 1:** Amino acid profiles (protein quality) of the eight tropical algae examined in this study and *Haliotis asinina* tissue (after [25], [57]).

Amino acid	*Hypnea*	*Ulva*	*Jania*	*Padina*	*Laurencia*	*Cystoseira*	*Sargassum*	*Asparagopsis*	*H. asinina* ^#^
lysine*	5.62	4.35^2^	5.11	4.48^2^	5.45^2^	4.73^2^	4.01^2^	4.32	8.60±0.23
threonine*	5.70	9.36	8.76	6.03	6.77	6.31	6.33	5.86	5.53±0.30
valine*	6.09	6.69	4.62	5.26	5.61	5.52	4.94	6.19	6.94±0.01
methionine*	2.37^1^	2.01^1^	1.95^1^	2.47^1^	2.31^1^	2.52^1^	2.62^1^	2.32^1^	5.82±0.01
Cysteine	3.40	2.01	5.35	1.24	1.98	1.26	1.39	2.13	0.60
isoleucine*	5.22	3.68	2.92	4.02	4.62	4.26	4.01	5.09	4.14±0.57
leucine*	7.99	6.69	5.84	7.73	7.59	7.89	7.41	8.25	8.69±0.25
Tyrosine	4.11	3.01	3.89	3.86	4.46	3.79	3.86	3.67	4.22
phenylalanine*	5.22	4.68	3.16	4.79	5.12	5.05	4.78	5.86	4.38±0.29
histidine*	1.42^2^	3.01	2.92	2.01	1.98	2.21	3.55	1.48^2^	3.04±0.51
arginine*	5.78	5.02	4.62^2^	5.10	5.78^2^	4.89	4.78^2^	7.15	8.36±0.58
tryptophan*	0.95	1.67	0.73	1.39	1.16	1.58	1.54	0.97	0.61±0.17
alanine	5.85	8.70	6.08	6.96	6.60	6.62	6.17	7.35	5.10±0.18
aspartic acid/asparagines**	11.63	12.37	9.73	15.15	12.38	13.41	12.81	12.24	8.35±0.50
glutamic acid/glutamine**	10.28	12.71	10.95	13.29	13.04	15.77	14.51	10.89	13.82±0.25
glycine	5.54	7.69	5.60	5.72	5.78	5.68	5.56	5.15	5.95±0.68
proline	6.25	0.00	9.98	5.41	3.96	3.63	6.94	5.15	4.79±0.05
serine	5.46	6.35	4.87	5.10	5.45	4.89	4.78	5.93	3.49±0.23
taurine	1.11	0.00	2.92	0.00	0.00	0.00	0.00	0.00	

Values are calculated using g amino acid 100 g^−1^ of protein.

* = Essential amino acid, ** = amino acids not distinguished and measured together, # = *H.asinina* requirement based on the mean values (±SE) of abalone tissue by [25] and [57].

1 = first limiting amino acid, ^2^ = second limiting amino acid.



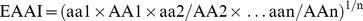



Where;

aa = A/E ratio in algae

AA = A/E ratio in *H.asinina*

n = number of EAA

A/E = EAA/total EAA (including cystine and tyrosine) × 100

### No-choice – Carrageenan Bound Diet

To evaluate the potential effects of morphology on the feeding preference hierarchy of *H. asinina*, a no-choice feeding assay was conducted using algae bound in carrageenan. Carrageenan was used as a binder for the artificial diet as it is also the structural polysaccharide in the highly preferred red alga *Hypnea* (see Results).

The eight species of algae were collected, washed of any epiphytes, freeze-dried and milled to a consistent powder (to <1 mm particle size using an Ika Werke MF 10 mill). Powdered algae was incorporated into carrageenan diets at 3.4% dried algae powder (the highest level possible to maintain binding strength), 4.6% carrageenan powder (Sigma, Type 1 commercial grade (predominantly κ, lesser amounts of λ carrageenan) and 92% distilled water. Powdered algae was initially mixed well with 26% of the distilled water in a beaker. Carrageenan was then mixed with the remaining 74% of the water in a separate beaker and microwaved on high for approximately 40–50 seconds until boiling. The mixture was stirred and microwaved until boiling point a further two times. The carrageenan mixture was then combined with the algae mixture and microwaved for 10–15 seconds on high. This mixture was rapidly poured into ice cube moulds to create cubes (35×20×20 mm). The cubes were set in a refrigerator.

The no-choice feeding assay for the carrageenan diets followed the same protocol as the natural diet no-choice feeding assay, placing treatment and control cubes into each container (n = 5 abalone per algal species). In addition, a control diet (carrageenan without algae) was included (n = 5 abalone). Animals ranging from 26 to 184 g (45 abalone in total) were distributed evenly to ensure that each treatment had a similar size range of abalone. Consumption rates for each replicate were measured (see Eq. 1) when more than ∼50% of the diet had been consumed or after 4 days. All carrageenan-bound diets maintained their integrity for the duration of the assay.

### Statistical Analysis

For choice assays, the consumption rate (g FW day^−1^) of each algae species within each replicate was ranked (lowest to highest). This ranked data was analysed using the non-parametric Friedman’s test of ranks, followed by Friedman’s post-hoc comparison [41]. Linear regressions were subsequently generated for abalone size (body weight) versus consumption rates for the total consumption rates of all algae in both choice assays as well as for each algal species independently.

For the no-choice feeding assays, consumption rates (g FW day^−1^) were analysed using analysis of covariance (ANCOVA) with algal diet as the fixed factor and abalone size (body weight) as the covariate for both natural and carrageenan-bound diet assays. ANCOVA assumptions of homogeneity of variance and normality were assessed using scatter plots and histograms of the residuals, respectively. Data were log transformed where appropriate. The ANCOVA assumption of linearity was assessed using the interaction terms of diet and abalone size for each analysis. Where no interaction existed, output from the reduced main effects model is reported. Diet treatments were compared using Tukey’s post-hoc comparisons, as appropriate. Both assays used a similar range of abalone sizes for each treatment. Linear regressions were also subsequently generated between algae protein content (adjusted for limiting amino acid) and mean consumption rates for each species (see Table 2 for input data).

**Table 2:** Consumption rates and the nutrition value of tropical algae for *Haliotis asinina*.

	*Hypnea*	*Ulva*	*Jania*	*Padina*	*Laurencia*	*Cystoseira*	*Sargassum*	*Asparagopsis*
**Wet: dry weight (DW)**	10.42^a^ ±0.3	10.15^a^ ±0.5	4.07^d^ ±0.1	4.66^cd^ ±0.1	8.95^a^ ±0.4	6.35^bc^ ±0.2	6.26^b^ ±0.5	5.32^c^±0.1
**DW consumption rate - natural** **diet, g 100 g^−1^ BW day^−1^)**	1.35^a^ ±0.14	0.87^a^ ±0.16	0.71^a^ ±0.09	0.19^b^ ±0.05	0.09^b^ ±0.01	0.08^b^ ±0.03	0.07^b^ ±0.03	0.09^b^ ±0.03
**DW consumption rate –** **carrageenan diet, g 100 g^−1^** **BW day^−1^)**	0.29^a^ ±0.02	0.29^a^ ±0.04	0.26^ab^ ±0.04	0.29^a^ ±0.02	0.15^b^ ±0.04	0.23^ab^ ±0.01	0.23^ab^ ±0.04	0.29^a^ ±0.03
**C:N**	10.55^e^ ±0.18	35.65^a^ ±0.91	28.47^b^ ±0.31	20.60^c^ ±0.45	16.15^d^ ±0.21	25.87^b^ ±1.40	30.36^ab^ ±2.9	7.34^f^ ±0.20
**Protein (g 100 g^−1^ DW)**	12.64	2.99	4.11	6.47	6.06	6.34	6.48	15.52
**EAAI**	0.82	0.83	0.79	0.86	0.85	0.86	0.85	0.82
**Effective Protein (g 100 g^−1^ DW)** **(adjusted for limiting AA)**	5.16	1.03	1.38	2.75	2.41	2.75	2.92	6.19
**Effective protein consumption** **rate (natural diet,** **mg 100 g^−1^ BW day^−1^)**	69.37^a^ ±7.25	8.99^bc^ ±1.65	9.76^bc^ ±1.22	5.14^cde^ ±1.39	2.26^def^ ±0.15	2.09^ef^ ±0.69	2.14^f^ ±0.75	5.29^bcd^±1.59

Data show means (±SE) for all indices. Dry weight consumption rates of algae (g 100 g^−1^ BW day^−1^) were calculated using wet:dry ratio (cf. Fig 2, fresh weight consumption of algal diets). Dry weight consumption rates of algae bound by carrageenan into artificial diets presented (cf. Fig. 3 fresh weight consumption of artificial diets). Carbon to nitrogen (C:N) ratio, protein content (g 100 g^−1^ DW: calculated as the sum of amino acids from Table 1), and essential amino acid index are presented. The effective protein content (g 100 g^−1^ DW: adjusted for the limiting amino acid from Table 1) and the effective protein consumption rate in the algal no-choice feeding assay (Fig. 2) are also presented. Common letter superscripts indicate no significant difference (ANOVA, Tukey’s HSD, p>0.05).

One-factor ANOVAs (analysis of variance) were used to compare wet: dry weight, nitrogen composition and the adjusted dry weight consumption rates. Data were log transformed to meet the assumption of homogeneity of variance where required (see Results). Post-hoc comparisons were made using Tukey’s HSD multiple comparisons.

## Results

### Choice assays – Natural Diet

**Figure 1:**
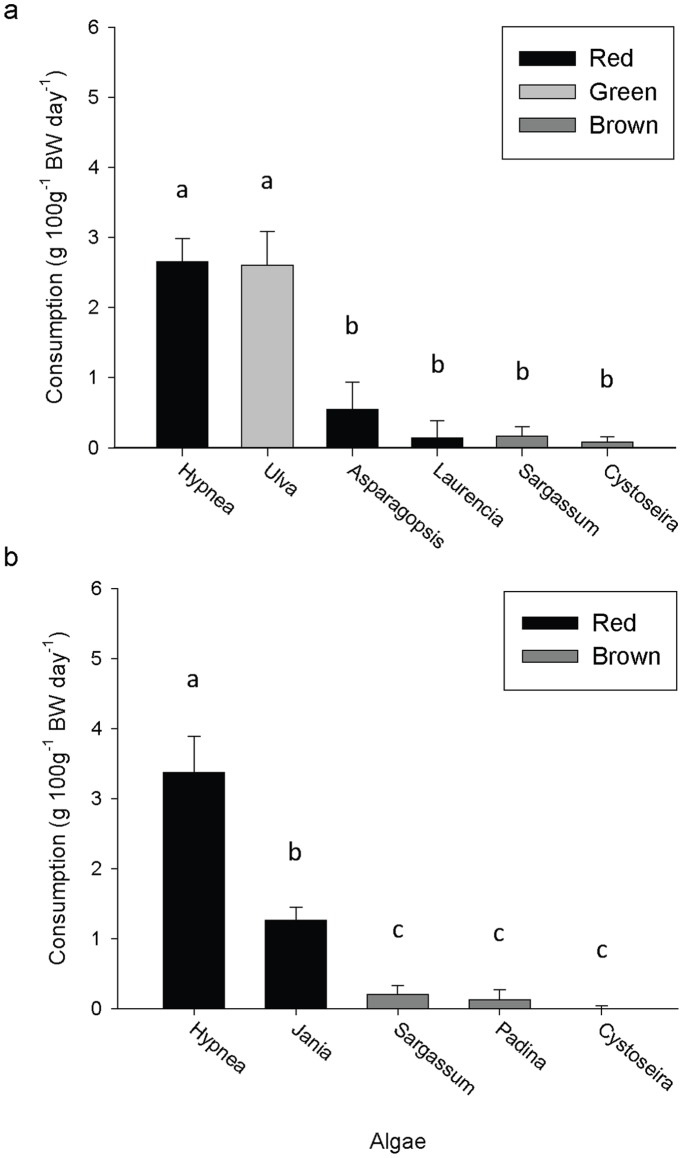
Feeding preferences of *H. asinina* in two separate multiple choice feeding assays (A & B). Data show mean (+SE) consumption rates of each species (g FW algae day^−1^) for red, brown and green algae standardised for abalone size (100g^−1^ BW). **A**. Preferences between 6 algal species (n = 11) with abalone size range of 35–80 g (mean = 54.26 g). **B**. Preferences between 5 algal species (n = 19) with a larger abalone size range; 29–184 g (mean = 95.63 g). Common letters above columns indicate no significant difference in preference between treatments for each assay (Friedman’s multiple comparisons, p>0.05).

*H. asinina* had clear and consistent preferences for a subset of algae in the choice assays (Friedman’s tests, p<0.001; Fig. 1a & b). *H. asinina* highly preferred *Hypnea* in both assays (Fig. 1a & b), consumed at the highest rates (2.65±0.33 and 3.37±0.52 g FW 100 g^−1^ BW day^−1^, respectively) and also preferred *Ulva* and *Jania* when they were provided in assay A & B, respectively (Fig. 1a & b). Three brown algae (*Sargassum, Padina* and *Cystoseira*) and two red algae (*Asparagospsis* and *Laurencia*) were consistently the least preferred species and correspondingly were consumed at very low rates (less than 0.54 g FW 100 g^−1^ BW day^−1^, Fig. 1a & b). Abalone consumed greater than 60% of one species of algae in less than 48 hours, with the exception of two replicates (one in each assay) which were excluded from statistical analysis.

In the first choice assay there were two distinct subsets of algae (Friedman’s test statistic = 30.10, p<0.001), where *Hypnea* and *Ulva* were consumed significantly more than all the other species *Asparagopsis*, *Laurencia*, *Sargassum* and *Cystoseira* (e.g. up to 38× more for *Hypnea* than *Cystoseira*). In the second choice assay there was greater distinction between algae, creating a clear hierarchy for the feeding preferences of *H. asinina* (Friedman’s test statistic = 52.98, p<0.001). *Hypnea* was again most preferred, followed by *Jania* (Fig. 1b). *Hypnea* was preferred more than all other species (Friedman’s post-hoc comparisons, p<0.05) and was consumed 3 times more than Jania and up to 26 times more than all of the other species *Padina*, *Sargassum* and *Cystoseira*. *Jania* was preferred more than *Padina*, *Sargassum* and *Cystoseira* (p<0.001) (Fig. 1b). The brown algae were consumed at consistently low rates and *Cystoseira* was untouched (Fig. 1b).

There was no relationship between abalone size and total consumption rates in the first choice feeding assay (R^2^ = 0.002, p = 0.901). However, the size range of abalone was small (35–80 g) and *Jania* was notably not present. The second choice assay used animals between 29–184 g. There was a significant positive relationship between abalone size and total consumption rates in the second choice feeding assay (R^2^ = 0.403, p = 0.005). This effect was driven primarily by the relationship between abalone size and *Jania* consumption (R^2^ = 0.572, p<0.001), as all other species had no pairwise relationship to consumption rates (p = 0.191–0.865). This included the highly preferred *Hypnea* (R^2^ = 0.033, p = 0.471).

### No-choice Feeding Assay – Natural Diet

**Figure 2:**
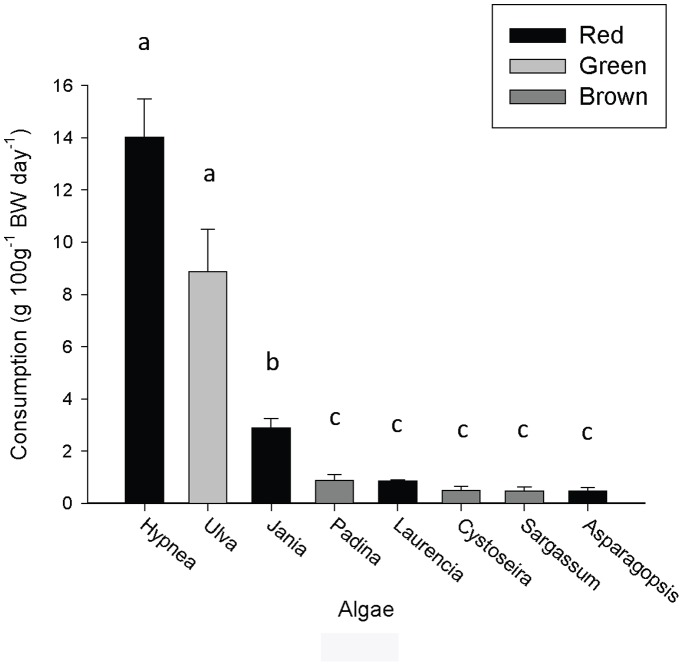
No-choice feeding assay for the 8 species of algae used in the previous choice assays. Data show mean (+SE) consumption rates (g FW algae day^−1^, n = 5) of algae per treatment standardised for abalone size (100g^−1^ BW). Abalone size ranged from 30–186 g (mean = 94.73 g). Common letters above columns indicate no significant difference (Tukey’s HSD, p>0.05).

Consumption rates in the no-choice feeding assay (Fig. 2) mirrored that of the preference hierarchy for *H. asinina* integrated across the two choice assays (Fig. 1a & b). The three most preferred algae in the choice feeding assays (*Hypnea, Ulva* and *Jania*) were consumed at higher rates than non-preferred species in the no-choice feeding assay (ANCOVA: log-transformed data, F_7,31_ = 49.58, p<0.001). *Hypnea* and *Ulva* were consumed more than *Jania* (7 and 4 times more, respectively), and *Jania* was consumed more than all remaining algae (3.5 to 7 times) (Tukey’s HSD, p<0.05). There were no differences amongst the five least consumed algae *Asparagopsis*, *Laurencia*, *Sargassum*, *Cystoseira* and *Padina*.

Consumption rates also increased with abalone size (ANCOVA: log transformed data, F_1,31_ = 11.27, p = 0.002). Mean consumption rates of the highly preferred species of algae (*Hypnea* and *Ulva* ) were considerably higher in the no-choice feeding assay (14.01 and 8.86 g FW 100 g^−1^ BW day^−1^, respectively) compared to the choice feeding assays (3.21 and 2.61 g FW 100 g^−1^ BW day^−1^, respectively). The consumption rates for *Hypnea* and *Ulva* in the no-choice feeding assay were also higher than the combined consumption rates of all algae in either of the choice feeding assays (6.55 and 5.51 g FW 100 g^−1^ BW day^−1 ^for choice assays A & B, respectively). *Hypnea* consumption rate was positively correlated to abalone size (R^2^ = 0.919, p = 0.01) in the no-choice assay, in contrast to the choice assays (see above). There were no trends between consumption rates and abalone size for any of the least preferred algae.

### Wet: Dry Weight

There was significant variation in the wet to dry ratio among the eight algal species (log transformed data: F_7,32_ = 71.68, p<0.001: Table 2). Wet to dry weight ratios were higher for *Hypnea, Ulva* and *Laurencia* (wet: dry; 8.95∶1–10.42∶1) compared to the five other species (*Asparagopsis*, *Jania, Cystoseira, Padina* and *Sargassum*; 4.07∶1–6.35∶1) (Tukey’s HSD; p<0.05: Table 2). *Jania* (4.07∶1) and *Padina* (4.66∶1) had the lowest wet to dry ratio (p<0.05). The preferred species had marked differences in the wet: dry ratio, for which *Hypnea* and *Ulva* had the two highest ratios and *Jania* the lowest. There was also a positive relationship between the wet weight consumption rate and the wet:dry ratio (R^2^ = 0.54 and p = 0.039), indicating higher consumption rates of algae with higher water contents on a fresh weight basis. However, there was effectively no change in the order of consumption rates when fresh weights were converted to dry weight consumption (ANCOVA: F_7,31_ = 38.39, p<0.001). *Hypnea* and *Ulva* (regardless of high wet: dry weight ratios) were still consumed at the highest rates, although *Jania* was no longer significantly different to *Hypnea* and *Ulva* (Table 2). All other comparisons remained the same. The positive influence of increasing abalone size on increased dry consumption rates remained (ANCOVA: log-transformed data, F_1,31_ = 11.27, p = 0.002).

### Compositional Analysis - Carbon and Nitrogen

The carbon to nitrogen ratios (C:N) varied from a low of 7.34∶1 in *Asparagopsis* and 10.55∶1 in *Hypnea*, to a high of 30.36∶1 in *Sargassum* and 35.65∶1 in *Ulva* (Table 2). There was a significant difference in the mean C:N ratios between the eight algal species (ANOVA: log-transformed data, F_7,16_ = 176.60, p<0.001). See Table 2 for pairwise comparisons.

Nitrogen content in the eight algae varied in a predictable and similar manner to the C:N ratio from a low of 0.4±0.01% in *Ulva* to a high of 2.5±0.11% in *Asparagopsis*. The nitrogen content for *Hypnea, Jania, Padina, Laurencia, Cystoseira* and *Sargassum* was 1.4±0.05% (±1SE), 0.5±0.01%, 1.1±0.04%, 1.2±0.05%, 1.2±0.06% and 0.9±0.05%, respectively. Carbon content ranged from a low of 14.3±0.4% for *Hypnea* to a high of 29.8±0.6% for *Cystoseira*. The carbon content for *Ulva, Jania, Padina, Laurencia, Sargassum* and *Asparagopsis* was 15.1±0.6%, 14.9±0.3%, 23.3±0.3%, 19.3±1.0%, 28.4±1.6% and 18.0±0.4%, respectively.

### Compositional Analysis - Protein Content and Amino Acid Profiles

Protein content varied substantially between species, with *Asparagopsis* having the highest protein content of 15.52 g 100 g^−1^ DW followed by *Hypnea* with 12.64 g 100 g^−1^ DW (Table 2). In contrast, *Jania* and *Ulva* had the lowest protein content at 4.11 g 100 g^−1^ DW and 2.99 g 100 g^−1^ DW, respectively. All algae species had similar essential amino acid indices (EAAIs), ranging from 0.79 in *Jania* to 0.86 in *Padina* and *Cystoseira* (see Table 2). When comparing the level of these amino acids to the requirements of *H. asinina* (based on the amino acid profile of abalone tissue: from Table 1), the first limiting amino acid was always methionine. The relative proportion of methionine for each algal species, calculated for *H. asinina* requirements, varied from a maximum of 45.1% in *Sargassum* to a minimum of 33.4% in *Jania* (Table 1). This equates to a 54.9–66.6% reduction in the potential protein for growth, assuming a monospecific diet and adjusting for the first limiting amino acid (“Effective Protein” in Table 2). Using this calculation, *Asparagopsis* and *Hypnea* still maintained the highest protein contents with reduced effective protein contents to 6.19 g 100 g^−1^ DW and 5.16 g 100 g^−1^ DW, respectively. *Ulva* and *Jania* maintained the lowest protein content with effective contents of 1.03 g 100 g^−1^ DW and 1.38 g 100 g^−1^ DW, respectively. For the two highest protein algae, *Asparagopsis* and *Hypnea*, a respective 22.0 and 14.6% increase in protein efficiency can be gained if methionine levels are increased to match the level of the next limiting amino acid (0.81 and 0.45 g kg^−1^ DW for *Asparagopsis* and *Hypnea*, respectively). The subsequent addition of histidine (second limiting amino acid) to these algae to a non-limiting level, 0.06 g kg^−1^ DW (+0.12 additional methionine) and 0.71 g kg^−1^ DW (+1.36 additional methionine) for *Asparagopsis* and *Hypnea*, respectively, results in a further increase in effective protein of 2.6% and 25.1% total for *Asparagopsis* and a further 37.0% and 56.9% total for *Hypnea*. *Hypnea* would have the highest effective protein level (8.3% vs. 7.9% for *Asparagopsis*) by supplementing methionine and histidine.

Overall, there was no relationship between the dry weight consumption rates and protein content (R^2^ = 0.002, p = 0.926) or to protein adjusted for the limiting amino acid (R^2^ = 0.003, p = 0.904), nor EAAI (R^2^ = 0.34, p = 0.129). Based on the combination of consumption rates and protein content, *Hypnea* provided *Haliotis asinina* with considerably higher protein (69.37 mg 100 g^−1^ BW day^−1^) than any of the other algae (Table 2). Protein intake per day varied significantly between algal diets in the natural diet no-choice feeding assay (ANCOVA: log-transformed data, F_7,30_ = 29.56, p<0.001). Despite its high consumption rates, *Ulva* provided *H. asinina* with daily protein levels similar to *Jania* and *Asparagopsis*, i.e. similar to a low consumption diet. *Cystoseira* provided the least protein per day (2.09 mg 100 g^−1^ BW day^−1^) (Table 2).

### No-choice Feeding Assay – Carrageenan Bound Diet

**Figure 3:**
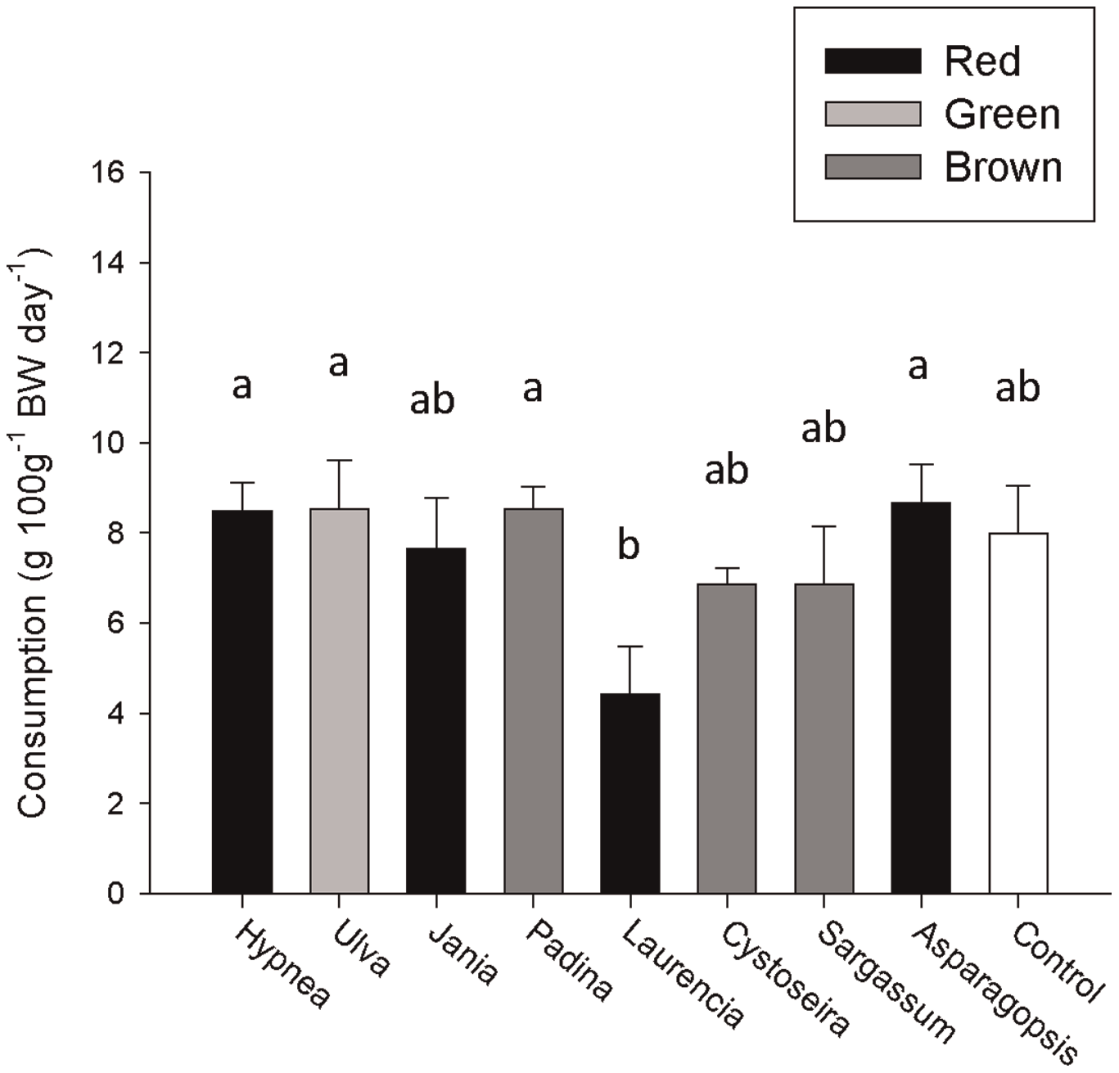
No-choice feeding assay of artificial diets comprised of dried algae and bound by carrageenan. Data show mean (+SE) consumption rates (g FW diet day^−1^, n = 5) of diets per treatment standardised for abalone size (100g^−1^ BW). Abalone size ranged from 26–184 g (mean = 89.63 g). Common letters above columns indicate no significant difference (Tukey’s HSD, p>0.05). Control diet (white bar, no algae) was also included in the formal analysis.

When dried algae were bound in carrageenan there remained a difference in mean consumption rate among the different algae (ANCOVA, F_8,35_ = 3.33, p = 0.006). There was no difference in consumption rates between the majority of species and the control diet (Fig. 3). However, *Laurencia* was consumed at a lower rate than *Hypnea*, *Ulva*, *Asparagopsis* and *Padina* (Tukey’s HSD, p<0.05). There was a positive correlation between abalone size and consumption rate (F_1,35_ = 121.93, p<0.001, R^2^ = 0.668, b = 0.05). Again there was no relationship between the effective protein content for each carrageenan bound diet and its consumption rate (R^2^ = 0.038, p = 0.617).

On a dry weight consumption rate basis, the dried algae consumed in the carrageenan bound diet no-choice assay tended to be lower for the preferred species compared to the natural diet no-choice assay (*Hypnea*: 0.29±0.02 and 1.34±0.14; *Ulva*: 0.29±0.04 and 0.87±0.16; *Jania*: 0.26±0.04 and 0.71±0.01 g DW 100 g^−1^ BW day^−1^ for carrageenan bound and natural diets, respectively). However, it was substantially higher for the non-preferred species (*Padina*: 0.29±0.02 and 0.19±0.05; *Laurencia*: 0.15±0.04 and 0.09±0.03; *Cystoseira*: 0.23±0.01 and 0.08±0.14; *Sargassum*: 0.23±0.04 and 0.07±0.03; *Asparagopsis*: 0.29±0.03 and 0.09±0.03 g DW 100 g^−1^ BW day^−1^ for carrageenan and whole plant, respectively).

## Discussion

*Haliotis asinina* had a distinct feeding preferences hierarchy amongst commonly available algae from shallow pan-tropical Indo-Pacific reefs. In summary, the red alga *Hypnea* and the green alga *Ulva* were highly preferred compared to *Asparagopsis, Laurencia, Jania, Cystoseira, Padina* and *Sargassum*. Consumption rates of algae in the no-choice assay mirrored that of the preference hierarchy observed in the choice assays, confirming distinct preferences between available tropical algae. Protein content varied substantially between species (from 3–15% of dry weight), however, there was no relationship between protein content and preference amongst algae, contrary to predictions for other species of abalone (see [2]). Furthermore, individual amino acids did not correlate with preferences including the first limiting amino acid for all algae - methionine. The most parsimonious explanation for feeding preference in *H. asinina* is that physical and chemical defences (or the lack thereof) have a far greater influence on the feeding preference hierarchy for *H. asinina*. This was further supported by the lack of clear preferences when carrageenan was used as a binder for the same algae in artificial diets, indicating that a wider selection of algae could be available for use in artificial diets than can be inferred from the whole-plant feeding preferences.

*H. asinina* has many attributes that make it attractive for commercial production, including high growth rates and high meat recovery [42]. However, little is understood about the feeding habits and nutrition of *H. asinina*. Most interpretation of dietary range and preference has come from the analyses of gut contents [43]. These data suggest that the natural diet consists predominately of red algae, most commonly the genera *Hypnea, Laurencia*, *Amphiroa* and *Coelothrix*. Of these *Hypnea* was most preferred in our study, yet *Laurencia* was one of the least preferred. It appears more likely that *Laurencia* was common in the guts of *H. asinina* because it is relatively more abundant in intense herbivory environments [44], or that its secondary metabolites influence digestibility [45], [46]. Regardless, *H. asinina* in our study had distinct preferences for algae, *Hypnea* and *Ulva*, that are neither chemically nor physically defended. This contrasts with temperate abalone and their preferences for tough, chemically defended brown algae in species from America (*H. rufescens*), Japan (*H. discua hannai* and *H. diversicolor supertexta*) and South Africa (*H. midae*) [7], [8], [12], [13] and chemically defended reds such as *Asparagopsis armata* in temperate Australian species (*H. rubra*, *H. laevigata* and *H. roei*) [5], [15].

The preference hierarchy of *H. asinina* showed no relationship to the total protein content or the effective protein content of the algae (based on the limiting amino acid content), nor to the EAAI of the algae, even though protein is the most important factor for abalone performance [23]. The two most preferred and consumed algae, *Hypnea* and *Ulva*, had high and low protein contents, respectively. Furthermore, *Asparagopsis* had the highest total protein content, but was one of the least preferred and consumed. In fact, all low preference algae in this study had higher protein contents than the highly preferred *Ulva*, making it unlikely that abalone preferentially select algae that are most nutritious. Feeding trials with brown algae and *H. rubra* have shown positive correlations between feeding preference hierarchies, performance and the level of digestible nitrogen and crude nitrogen [2], [18], and similar data also exists for other gastropods [19]. However, in all of these studies feeding preferences were negatively correlated with polyphenolic content [2], [18], [19]. These data suggest that preferences can be driven by digestible rather than total nitrogen content, and, at least for temperate brown algae, this relationship is strongly governed by the level of anti-nutritional phenolic compounds [4], [18]. However, tropical brown algae such as *Sargassum* [47], [48] and *Cystoseira* [49] have low contents of phenolics. Therefore the low consumption rates of brown algae in both no-choice and preference experiments indicate that *Sargassum* and *Cystoseira* most likely have physical limitations for feeding, especially when alternative diets are accessible. The toughness of algae can greatly influence feeding behaviour in abalone [14], [16] and may explain the low preference for the calcified *Jania* and *Padina* in our study. More specifically, calcification of diets have been shown to deter feeding by herbivorous fish [50] and may also be the likely reason behind the lower consumption rates of *Jania* by smaller abalone in the second choice feeding assay. In contrast, a suite of bioactive halogenated metabolites are established feeding deterrents in both low preference red algae *Asparagopsis* [15] and *Laurencia* [46] which have demonstrated effects on abalone and other molluscs. Our study, and others (see also [21]), suggest that the apparent feeding preference may be determined by a compromise between nutritive values and deterrence effects (physical and chemical) of the potential species available in the environment. Thus it is important to emphasise that our data demonstrate that any chemical and physical defences in these tropical algae, if present, can be quenched by incorporating biomass into artificial diets.

The feeding preference hierarchy for the eight natural algae was neutralised when the algae were bound in a carrageenan matrix, and the high consumption rate of the control diet (without algae) suggests that carrageenan may be a feeding attractant. The incorporation of algae into artificial diets can stimulate consumption in abalone [51] and other marine herbivores, including sea urchins [52]. A stimulant effect of carrageenan for *H. asinina* would also explain the increased consumption of *Asparagopsis* despite high concentrations of chemical defence [15] and may mask phenolic compounds in the three brown algae [4], [18]. The increases in the consumption rate of *Jania*, and to some extent the brown algae *Padina* and *Sargassum*, may in part be due to the reduced efficacy of physical defences in these algae. Carrageenan is a structural polysaccharide that makes up a large proportion of the dry weight of *Hypnea* (48% DW; [53]). Furthermore, we observed that *H. asinina* responded instantly to the addition of *Hypnea* into tanks, moving rapidly around the tank. While this response was not quantified, it did not occur for any other algal species. The significantly lower consumption rate of the *Laurencia* diet suggests that the bioactive secondary metabolites in this genus [45] remain partially effective in the carrageenan matrix. Longer term performance trials should therefore evaluate whether the elevated consumption of protein-rich chemically defended algae in artificial diets, specifically *Asparagopsis*, have an adverse effect on abalone growth and survival through mechanisms similar to that established for brown algal phenolics [18]. Furthermore, *Asparagopsis* species have the highest recorded aquaculture productivity rates for algae [54], which makes them an attractive and sustainable option for cost-effective alternative feed sources.

Our study examined a variety of algae based on nutrient composition and feeding preference by *H. asinina* and found that the red algae *Hypnea* and *Asparagopsis* had the highest protein content, and that *Hypnea* was the most preferred and highly consumed of all tested species – supporting it as a suitable candidate species for use in the development of abalone feeds. However, no broader link between algal nutrition and the preference hierarchy for *H. asinina* was found, demonstrating that preference based on nutrition is not a paradigm for all abalone. Instead, the overriding factors influencing the feeding preferences of *H. asinina* are more likely the physical and chemical defences of algae, both of which can be diminished through the use of carrageenan bound diets. Although this result means that algae could instead be selected based on nutritional value rather than feeding preferences, these diets would likely be limited by methionine, the calculated first limiting amino acid for all algae in this study. Thus mixed algal diets may only reach an optimal amino acid profile if methionine is supplemented in diet formulation, followed then by histidine for the high protein target species *Hypnea* and *Asparagopsis*. With these two amino acids the effective protein would increase by a total of 25% and 57% for *Asparagopsis* and *Hypnea*, respectively. The addition of such free essential amino acids (methionine, histidine, lysine and arginine) into diets is a common practice for many agriculture animals such as pigs [55] as well as for aquaculture animals such as prawns [56]. Our study suggests that such practices would also be beneficial for abalone when fed algae-based diets.
